# Multiple sclerosis: 2026 update

**DOI:** 10.17879/freeneuropathology-2026-9705

**Published:** 2026-05-31

**Authors:** Luisa Klotz, Mike P. Wattjes, Tanja Kuhlmann

**Affiliations:** 1 Department of Neurology, University Hospital Münster, Münster, Germany; 2 Department of Neuroradiology, Charité - Universitätsmedizin Berlin, Corporate Member of Freie Universität Berlin, Humboldt-Universität zu Berlin, Berlin, Germany; 3 Institute of Neuropathology, University Hospital Münster, Münster, Germany

**Keywords:** Multiple sclerosis, Aging and cellular senescence, Tissue resident memory cells, Biomarkers, Clinical trials

## Abstract

Multiple sclerosis (MS) is the inflammatory-demyelinating disease of the CNS with the highest prevalence. Despite significant progress in reducing relapse activity by immunomodulatory or immunosuppressive treatments, there are ongoing challenges in understanding its initiation and predicting disease evolution. Furthermore, since we only partially understand the mechanisms driving disease progression, its successful treatment represents a major challenge. Therefore, this review article summarizes key research findings from the last 18 months, covering advances in the understanding of pathophysiology including the effects of aging on glial and immune cells as well as the functional role of tissue-resident memory cells. Additionally, we discuss advances in imaging technologies and biomarkers as well as the use of AI to predict disease evolution. Finally, we report the most recent findings from clinical trials targeting immune cells within the CNS.

## Introduction

Multiple sclerosis (MS) is the most frequently diagnosed chronic inflammatory demyelinating disease of the central nervous system leading to long-term disability in young adults. It is characterized by a complex immune-mediated and neurodegenerative pathology [1]. Although high-efficacy immunomodulatory therapeutic interventions have significantly advanced the management of focal inflammatory activity, the field faces persistent challenges regarding disease initiation and the unpredictable nature of clinical trajectories. In recent years, progression independent of relapse activity (PIRA) has emerged as an important new concept, and it has been shown to be a major driver of accumulation of disability even when focal inflammatory activity is sufficiently controlled by high efficacy disease-modifying therapies (DMTs) [2,3]. However, the underlying disease mechanisms contributing to PIRA are still poorly understood. Advanced data analytics of existing high quality data sets employing AI and machine learning may help to improve our understanding of individual disease trajectories and thus promote the development of prognostic biomarkers. Furthermore, emerging insights into the pathogenic roles of myeloid cells, tissue resident memory T cells and B cells are currently reshaping the therapeutic landscape, fostering the development of novel agents such as Bruton tyrosine kinase inhibitors aimed at mitigating disease progression [4,5].

**Figure 1: Immune cells reach the CNS via the blood and the choroid plexus:**
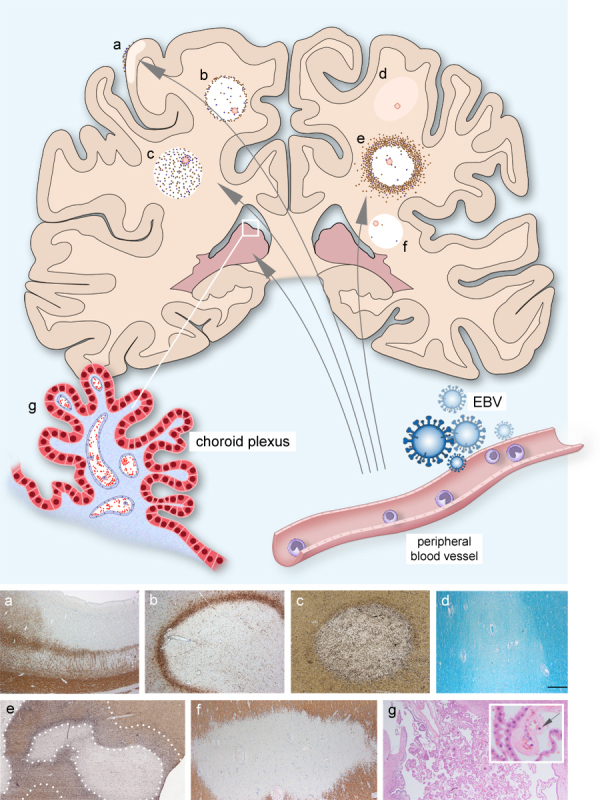
In the schematic illustration different MS pathologies caused by invading immune cells are depicted. The pictures at the bottom depict a cortical lesion **(a)** (staining for the myelin marker MBP), (b) a mixed active / inactive lesion (= chronic active lesion, staining for the myeloid cell marker HLADR), **(c)** an active lesions (double immunohistochemistry for the myelin marker PLP (brown) and the myeloid cell marker HLADR (black)), **(d)** a remyelinated lesion (LFB-PAS), **(e)** a broad rim lesion (double immunohistochemistry for the myelin marker PLP (brown) and the myeloid cell marker HLADR (black)), **(f)** an inactive lesion (immunohistochemistry for the myelin marker MBP) and **(g)** choroid plexus; the arrow in the inserts labels a lymphocyte migrating through the vessel wall.

Based on these considerations, the aim of this review article is to focus on key publications published in the last 18 months that provide new insights into MS pathophysiology including EBV and ageing, disease prediction and trajectories as well as treatment strategies (**Figure 1**).

## Topic 1: New findings from MS animal models potentially relevant for the human disease

In the year 2025, a number of relevant experimental in vivo studies have been published. In this review article we selected and summarize articles, we considered to be of special interest. These studies address unresolved questions regarding the pathophysiology of MS, i.e. the molecular mechanisms underlying lesion initiation and formation, macrophage polarization and functions as well as neurodegeneration.

Ling and colleagues combined histological, single-nucleus RNA (snRNA) sequencing, spatial transcriptomics and longitudinal imaging to dissect the cellular and molecular mechanisms underlying lesion formation in a marmoset model of multiple sclerosis [6]. A newly established MRI lesion model including the ratio of signal intensities on proton density-weighted images to T1 relaxation times aimed to represent the phase of high cellularity preceding myelin damage. Serial MRI retrospectively estimated lesion age by mapping the temporal sequences of cellular changes and correlating them with results from snRNA sequencing and spatial transcriptomics. The authors observed increased signaling from perivascular astrocytes and periventricular ependymal cells before lesion development. In addition, they identified SERPINE1+ astrocytes as a secretory hub underlying lesion initiation in these CNS regions. Early steps of lesion formation included proliferation and diversification of microglia and oligodendroglial progenitor cells followed by invasion of monocytes at the lesion center and persisting lymphocyte infiltrates at the lesion border. Already 10 days after initiation of lesion formation, reparative molecular signatures at the lesion edge were detected. Based on analyses of receptor-ligand interactions, the authors described global shifts in cellular connectivity allowing the identification of potential new drug candidates. The EAE marmoset model, combining longitudinal imaging with histological and spatial transcriptomic analyses, provides for the first time temporally resolved insight into cellular and molecular lesion dynamics. However, whether EAE more faithfully models multiple sclerosis or MOGAD pathophysiology continues to be debated.

Myeloid cells play a pivotal role in lesion formation of demyelinating lesions in MS and its experimental animal models. Their plasticity and context-dependent activation states are shaped by the tissue environment. However, the molecular cues triggering in vivo the switch from one macrophage state to another, for example from lesion-promoting to a lesion-resolving state are only partly understood. De la Rosa and colleagues established an in vivo CRISPR screening pipeline using a genetically editable hematopoietic progenitor cell line to test the functional role of more than 100 genes for macrophage functions in a single experiment [7]. The authors confirmed IFNγ, TNF, GM-CSF and TGFβ as key drivers of macrophage polarization in different tissue compartments in experimental autoimmune encephalomyelitis (EAE) models and presumably also in MS lesions. Interestingly, IL-4, IL-10 and IL-13 which modulate myeloid cell polarization in vitro, were of less importance in the EAE model used in this study. Furthermore, the authors also dissected new roles for TGFBR1 and TGFβ. Whereas TGFBR1 contributes to lipid efflux in macrophages, TGFβ regulates the migration of macrophages from the parenchyma to the meninges. In summary, the authors did not only develop an in vivo CRISPR screening approach to dissect the signaling cues regulating macrophage states, but also provided new insight into myeloid cell regulation in MS animal models and presumably also MS.

The molecular mechanisms driving neuronal dysfunction and loss in MS are still not fully understood. However, modern -omics analyses of MS tissue and complementary animal models continuously contribute to the identification of intracellular signaling pathways leading to neuronal loss. Recent studies by Manuel Friese and his team further highlight IFNγ released by immune cells as an important driver of neurodegeneration in MS [8]. IFNγ induces expression of the stimulator of interferon genes (STING), which is elevated in neurons in MS and EAE lesions. STING activation occurs after detachment from stromal interaction molecule 1 (STIM1), triggered by glutamate excitotoxicity. This leads to autophagic degradation of glutathione peroxidase 4 (GPX4), impaired redox homeostasis, and ferroptosis [8]. IFNγ signaling also increased expression of the immunoproteasome subunit proteasome 20S beta 8 (PSMB8), reduced proteasome activity, and caused accumulation of phosphofructo-2-kinase/fructose-2,6-bisphosphatase 3 (PFKFB3) in neurons. This enhanced glycolysis, reduced pentose phosphate pathway activity, promoted oxidative injury, and again resulted in ferroptosis. Notably, PSMB8 is upregulated in neurons in experimental MS models and in cortical grey matter MS lesions [9]. These findings emphasize the role of metabolic dysfunction in neuronal loss, and suggest that efficient elimination of inflammatory mediators and immune cells within the CNS may prevent activation of IFNγ-driven intraneuronal injury pathways.

## Topic 2: New pre-clinical models to investigate MS-related pathomechanisms and new treatment approaches

*Human iPSC derived models:* The search for pro-remyelinating or directly neuroprotective treatments is hampered by the lack of pre-clinical in vitro and/or in vivo models faithfully recapitulating the pathology and clinical characteristics of MS. In recent years, pluripotent stem cell derived 2D and 3D cultures came more and more into focus as a way to address the species differences between humans and the different experimental animal models, which limits at least partially the translatability from animal models to human diseases. Earlier studies mostly used stem cell derived monocultures (e.g. neurons or oligodendrocytes) or co-cultures. More recently, a number of articles were published, in which brain organoids were applied to either investigate disease pathomechanisms or to test new pharmacological approaches. Kazakou and colleagues used iPSC derived oligodendroglial monocultures or brain organoids to study the effects of metformin on CNS cells [10]. Metformin is currently under investigation in a number of clinical trials to test its pro-myelinating effects. However, it is also under investigation as a direct neuroprotective agent in MS and neurodegenerative diseases including Parkinson's and Alzheimer's diseases. One assumed mode of action of metformin is the rejuvenation of oligodendroglial progenitor cells which should counteract the age associated decline of differentiation and myelination potential of adult OPC [11,12]. Kazakou and colleagues demonstrated that metformin increased the differentiation of embryonic stem cell derived oligodendrocytes in monocultures as well as brain organoids suggesting that this effect is mediated via metformin's effects on mitochondria [10].

Major obstacles for the use of brain organoids as MS disease model are the lack of microglial and sufficient myelination. Therefore, the aim of the study by Lange and colleagues was to further optimize human brain organoids as a disease model for MS by integrating microglia and induce demyelination using lysolecithin [13]. They demonstrated that clemastine, XAV939, and BQ3020, all molecules that have been shown previously to promote oligodendroglial differentiation and/or (re-)myelination in several preclinical models, also enhanced the formation of new myelin sheaths in brain organoids. However, although these organoids contained microglia it is questionable whether this is sufficient to model the inflammatory environment in MS lesions. Stanton and colleagues further advanced human brain organoids by developing so-called multicellular integrated brains containing neurons, oligodendrocytes, astrocytes, microglia, pericytes and endothelial cells [14]. They demonstrate the usefulness of this model to study Alzheimer’s disease pathologies associated with *APOE4* genetic risk [14]. However, whether these brain organoids can be used to model MS pathology remains to be determined. An additional limitations of all iPSC derived models is the loss of age and / or disease associated epigenetic modifications due to reprogramming (see also Topic 4).

*New animal models:* The search for new pre-clinical animal models representing key aspects of MS pathology and allowing the testing of potential pro-myelinating or neuroprotective drugs is ongoing. The use of non-human monkeys, such as marmosets is one approach (see also above). Sarrazin and colleagues induced demyelinating lesions in optic nerves of marmosets using lysolecithin [15]. In this model, remyelination was markedly impaired and associated with progressive neuronal degeneration, and visual dysfunction, thus recapitulating some aspects also observed in MS [15]. Importantly, the model allows assessment of functional consequences of pharmacological treatments using visual evoked potential, optical coherence tomography, and electroretinogram. In their recently published study, the authors now used the model to investigate the remyelination potential of clemastine fumarate [16]. Their findings show that clemastine was able to improve remyelination in the optic nerves to a certain degree; however, it did not reduce axonal loss and had no significant effect on the functional read-outs [16]. An alternative approach to improve the translatability of findings from animal models to human diseases, is the use of humanized mouse models. Kazakou and colleagues transplanted hESC-derived PDGFRA^+^ OPCs into the corpus callosum of ^-/-^*Rag2:Shi/Shi* P2-P4 mice, which lack the myelin protein MBP. They observed a slight, but significant increase in the number of myelinated axons after treatment of the mice with metformin for 21 days [10].

*Human iPSC derived CNS cells and humanized mouse models for drug discovery:* Cacem and colleagues developed a drug discovery pipeline including in silico screening, rodent in vitro assays, human iPSC derived 2D and 3D in vitro assays as well as different mouse models including a humanized mouse model. Using this pipeline, they screened a library of 1500 repurposed drugs to identify compounds with pro-remyelinating and neuroprotective effects [17]. The H3 antagonist bavisant displayed pro-remyelinating and/or neuroprotective effects in all animal models including the humanized mouse model. In the latter, bavisant increased significantly the number of axons remyelinated by human oligodendrocytes.

In summary, these studies demonstrate significant efforts to further optimize the existing preclinical in vitro and in vivo models for MS. However, the scientific community still faces significant challenges concerning the accessibility and practical applicability of these models. Furthermore, significant additional optimization is required to be able to use human brain organoids in high-throughput drug screens.

## Topic 3: The role of EBV in MS

In recent years, several papers have fueled the scientific interest into the role of EBV and MS. The seminal paper by Bjornevik and colleagues for example demonstrated a 32-fold increased MS risk after EBV infection in adolescence [18]. Additionally, the detection of molecular mimicry between the EBV transcription factor EBNA1 and the central nervous system protein glial cell adhesion molecule (GlialCAM) and cross-reactive antibodies in CSF and blood from people with MS further supported the link between EBV infection and MS. [19]. Several very recent studies have now gathered novel evidence further substantiating the connection between EBV and MS. Thomas and colleagues identified anoctamin-2 (ANO2) as a major autoimmune target with anoctamin-specific T cells being present in approximately 57 % of untreated MS patients and cross-reacting with EBNA1, a prominent EBV-derived antigen [20]. In an animal model of CNS autoimmunity, anoctamin-specific T cells aggravated brain inflammation, with anoctamin-specific T cells exhibiting pathogenic and cytotoxic features and infiltrating ANO2-expressing brain regions enforcing blood-brain-barrier (BBB) leakage. It should be noted, however, that neither anoctamin-peptide-immunization alone nor isolated transfer of anoctamin-specific T cells were capable of eliciting CNS autoimmunity in this animal model.

Additionally, the molecular mimicry concept is stressed by another paper by Wang et al showing that the HLA-DR15 risk allele specifically alters the B cell immunopeptidome to present EBV peptides to T cells that structurally mimic myelin antigens [21]. These "myelin-mimicking" peptides were enriched in the peripheral blood and CSF of MS patients. This observation supports the notion that EBV infection-related release of such antigens in the context of a particular HLA background facilitates not only the generation but also the presentation of such antigens to T cells and hence generating antigen-specific T cell responses with a high potential of cross-reacting with autoantigens. Further supporting the pathogenic role of EBV specific T cells, Läderach et al utilize single cell sequencing to reveal that EBV-specific T cells exhibit a signature for CNS homing, inflammation and cytotoxicity. In fact, these cells are found both in the CSF of MS patients as well as MS brain lesions [22]. Finally, Kim et al characterize B cells as potential engine of this antigen-specific autoimmune process, in particular focusing on their capture and presentation of CNS autoantigens to T cells [23]. In this context, the researchers revealed an interplay between two key risk factors for MS, i.e. EBV and the HLA allele HLA-DR15, as they found that distinct myelin peptides are taken up by B cells through their B cell receptors and subsequently presented to T cells. This cognate interaction was found to be particularly present in HLA-DR15 carriers. An interesting functional link to EBV is given by the observation that EBV abrogates B cell death upon antigen-uptake while lacking T cell help, as EBV-elicited LAMP1 expression serves as a surrogate for T cell help via CD40L, therefore preventing B cell death and thus promoting antigen-specific reactivation of memory CD4 T cells by antigen-specific B cells. Together, these new research avenues provide more pieces of evidence to our understanding of the link between EBV and MS, in particular substantiating the relevance of molecular mimicry between EBV peptides and CNS antigens, as well as explaining the mechanisms of amplified MS risk in the combination of HLA-DR15 – the strongest known genetic risk factor for MS – and EBV. Further studies should substantiate these new lines of evidence by illustrating their involvement into the initiation and/or propagation of CNS inflammation.

## Topic 4: Consequences of aging and cellular senescence in MS

Chronological age is strongly associated with the clinical course of multiple sclerosis. Whereas young patients almost exclusively present with relapsing remitting disease, individuals with later disease onset disease are rather characterized by a more rapid development of permanent disability [24]. However, the cellular and molecular mechanisms in immune and neural cells contributing to these age-associated differences are only poorly understood. Many aspects of T-cell aging for example are conserved in MS; however older MS patients harbored increased frequencies of CD4 T cells with an activated and cytotoxic effector profiles [25]. Interestingly, older MS individuals also displayed decreased expression of T-cell co-inhibitory receptor CTLA-4, and increased B-cell costimulatory molecule expression, which may contribute to the aberrant frequency of activated CD4+ T cells. However, to disentangle the effect of age and immunosenescence on the disease course is challenging due to the complex interplay between age and other factors, such as sex, viral infections, co-morbidities and treatment [26]. To study the age associated changes in glial and neuronal cells is even more challenging due to the limited access of tissue samples from healthy individuals and pwMS. To circumvent this obstacle, Windener et al directly converted fibroblasts from young (22 to 32 years) and older donors (65 to 71 years) into oligodendrocytes (dchiOL) [27]. Whereas reprogramming of somatic cells into stem cells is associated with the erasure of many age-associated epigenetic marks, direct conversion of fibroblasts into the desired cell types preserves the epigenetic age of the donor cells. DchiOL from older donors displayed an impaired terminal differentiation, increased ROS production and upregulation of cellular senescence markers. Furthermore, dchiOL had an age- and cell type specific transcriptome and methylation signature, which was different from the donor fibroblasts. The epigenetic aging signatures of dchiOL and human white matter tissue samples correlated and epigenetic aging was accelerated in the normal appearing white matter (NAWM) of MS brain donors compared to white matter from healthy controls. Interestingly, supernatants from pro-inflammatory microglia could induce some of the age-associated changes in young dchiOL, suggesting that the inflammatory environment in the brain of pwMS may contribute to the accelerated epigenetic aging observed in MS tissue samples. Fagiani and colleagues choose a different strategy [28]. Using single cell RNA sequencing and spatial transcriptomics they observed a higher frequency of glial cells with expression of some senescence markers (termed senescent-like glial cells) in the white matter and chronic active lesions from pwMS compared to tissue samples from healthy individuals. Interestingly, a radial pattern of senescent-like changes from lesion cores to periplaque white matter was found, which was associated with a change in cell-cell communication. Exposure of human iPSC derived brain organoids to CSF from pwMS resulted in an increase in senescent-like cells. Microglia were the most affected cell type, which might be not surprising since the senescent signatures contained a high number of immune-related genes. Treatment with ibudilast, MR-α-lipoic acid and tolebrutinib reduced the number of senescent-like cells induced by MS-CSF exposure. To further support the notion that inflammation induces cellular senescence of glial cells in MS, the authors estimated the brain age gap (defined as the chronological age subtracted from the predicted brain-age) in pwMS using publicly available transcriptomic signatures and MRI. Both approaches confirmed a higher age-gap in pwMS and this was especially obvious in either inflamed lesion areas or pwMS with a higher number of paramagnetic rim lesions (PRLs) and higher lesion load.

The results of these studies underscore the relevance of age-associated cellular senescence not only for the immune system but also for CNS-intrinsic cells. The -omics technologies nowadays available will enable the further disentanglement of the complex relationships between physiological aging, inflammation and CNS intrinsic cells, also in a disease context.

## Topic 5: Choroid plexus in MS

The routes inflammatory cells take to enter the brain are still a matter of debate. The choroid plexus (CP) has come into focus as one key entrance route. Postmortem studies revealed an increased number of lymphocytes and myeloid cells in CP of pwMS [29,30]. Fleischer and colleagues observed that CP volume correlated positively with CSF albumin levels and negatively with cortical thickness and cognitive performance. Furthermore, high efficacy treatment prevents CP enlargement [31]. The same group now extended their investigations and performed a prospective multi-center, longitudinal study including 891 pwMS undergoing high-resolution 3T brain MRI, soluble neurofilament level (sNfl) measurements and clinical assessment over six years to investigate the prognostic value of CP volume and its longitudinal change for features associated with neurodegeneration, such as sNfl and disability progression. High CP volume resulted in a 1.8-fold increased risk of disability worsening and a 2.7-fold increased risk of progression independent of relapse activity [32]. However, the question remains open whether CP enlargement and the increased number of inflammatory cells in the CP are causally contributing to neurodegeneration or whether it is an epiphenomenon of an activated immune system and/or impaired Brain-CSF barrier which results in both, increased inflammatory infiltrates in CP and meninges.

## Topic 6: Tissue resident memory cells in MS

Tissue resident memory cells (TRM) are long-lived T cells acting as permanent tissue sentinels to identify foreign threats and initiate fast antigen-specific immune responses. Their accumulation in the CNS in the context of MS has already been observed before, and a pathophysiological relevance of this population has been assumed. Several publications from 2025 have now contributed experimental evidence of their role in driving local inflammation within the CNS. A study by Pignata and colleagues described the presence of bona fide TRM cells characterized by signature markers including CXCR6, CD69, P2RX7 and CD49a in a chronic EAE model as well as in postmortem human brain tissue, in particular in inflammatory demyelinated lesions [33]. Single cell transcriptomic analysis revealed that these TRM cells are clonally expanded and functionally heterogenous with distinct proinflammatory subsets. Notably, ablation of TRM cells within the CNS while simultaneously abrogating their replenishment from the periphery decreased disease severity during the chronic phase of EAE, providing direct evidence of their pathophysiologic relevance and shedding light into their role as novel therapeutic target. The alleviation of neurological deficits in these animals was accompanied by reduced microglial and astrocyte activation, which suggests that TRM cells contribute to the chronification of local inflammatory processes. An interesting open question remains whether TRM cells in humans may be a critical contributor to the persistence of inflammation in MS lesions in the context of disease progression. With regard to the evolution of TRM cells in MS, Hsiao and colleagues demonstrated in a systematic analysis of T cell phenotypes in distinct CNS compartments from human post mortem brain donors a predominant effector memory-like phenotype in the CSF as well as in the white matter compartment [34]. In particular the border-associated T cells exhibit features of TRM cells, and at least in vitro, direct interaction of T cells with brain endothelial cells elicits such phenotypic changes, such as progressive acquisition of CD49a, CD103 and ultimately CD69. Together, this study supports a concept of controlled acquisition of TRM cell-associated traits at the meninges and choroid plexus, which ultimately gives rise to the resident T cells that populate the human brain. A still unresolved question is whether in MS, generation of TRM cells is a normal feature just affecting T cell clones recognizing autoantigens, or whether TRM development itself may be altered or promoted compared to healthy individuals, and which mechanisms may underlie this process.

Another study by Feng et al used spatial transcriptomics combined with single nucleus sequencing to investigate the interplay between T cells and local immune cells in human chronic active MS lesions [35]. They observed an enrichment of tissue resident memory CD8 T cells in the lesion rims. These CD8+ T cell niches, which were associated with inflamed microglial cells, displayed an interferon signature accompanied by upregulated lipid metabolism. In accompanying animal experiments, they could show that dysfunctional lipid recycling in microglia resulted in exacerbated disease accompanied by a significant increase in CD4 and CD8 T cells into the inflamed CNS, whereas pharmacological stimulation of lipid efflux reduced disease severity while decreasing the accumulation of T cells in the CNS. These data give rise to the idea that the cross-talk between CD8 TRM and microglial cells may perpetuate microglial activation at the lesion borders; however, this needs to be further addressed experimentally.

## Topic 7: Cellular and molecular mechanisms driving MS disease biology

The mechanisms driving lesion initiation are still only poorly understood. Whereas Lin and colleagues used the marmoset model to experimentally investigate this question (see above) [6], Abdalhak and colleagues made use of the Department of Defense serum repository, where samples were available over a period of several years before clinical disease onset from 134 people with MS, and they leveraged proteomics to detect the sequence of injury to different CNS cellular components [36]. Interestingly, MOG as marker for myelin injury was found to be elevated already 7 years before clinical disease onset, while neurofilament (NEFL) elevation as a proxy of neuroaxonal damage became evident 1 year later. GFAP levels as markers for astrocyte involvement differed between healthy donors and pwMS only from disease onset on. These findings have several implications: Firstly, they demonstrate how early CNS pathophysiology precedes clinical onset of MS and thus further support the currently prevailing concept of a biological definition of MS rather than a clinical one. Secondly, they provide evidence of demyelination occurring prior to neuroaxonal injury thus strengthening the notion of MS as primarily demyelinating disease, and thirdly, the late involvement of astrocytes argues against a crucial role of this cell population in the incipient phase of disease.

Chronic active lesions, also called mixed active/inactive lesion have come into focus during recent years, since combined histology and imaging studies suggest, that a higher number of chronic active lesions with iron depositions are associated with a more rapid disease course [38]. To further deepen our understanding which pathological patterns are associated with a more severe disease course Klotz, Smolders and colleagues investigated the histopathological characteristics of MS brain donors with opposing disease trajectories [39]. Rapid disability progression was associated with a higher lesion load in brain stem and spinal cord, a greater proportion of active and chronic active lesions, increased lesion burden in the cortical grey matter, and a lower proportion of extensively remyelinated lesions. Interestingly, a subset of clinically rapidly progressing cases exhibited lesions with a broad rim of myeloid cells, defined as a ≥ 1 mm-wide myeloid cell rim and termed broad rim lesions (BRL). Using spatial transcriptomics and GO term analysis of myeloid cells, the authors identified transcriptomic signatures shared by active, chronic active and BRL lesions consisting of genes related to antigen presentation and T and B cell; further supporting the notion that reciprocal interactions between myeloid cells and lymphocytes contributes to the perpetuation of inflammation in MS lesions [6,40]. In contrast, BRL-specific myeloid cell gene signatures indicated enhanced protein turnover, pro-inflammatory cytokine production, apoptosis and myeloid cell migration suggesting elevated innate immune activity linked to endoplasmic reticulum (ER) stress and the unfolded protein response (UPR). Using Positron-Emission-Tomography (PET) using TSPO tracers defined (rBRL) were identified and the proportion of rBRL strongly correlated with the TSPO-PET signal in normal-appearing white matter, further suggesting an exaggerated innate immune response.

These studies demonstrate the importance of well characterized body fluid and tissue collections to dissect the cellular and molecular mechanisms driving disease biology and dynamics of MS. They further underscore the importance of CNS intrinsic neuroinflammation for the disease and presumably disease progression.

## Topic 8: Paraclinical markers of MS

### Imaging chronic inflammation and synaptic loss

Imaging is a very important paraclinical tool for MS diagnosis and disease monitoring. However, there is still an unmet need of MRI markers that specifically address disease mechanisms beyond acute focal inflammation. In particular, imaging of chronic inflammation remains challenging and has not been implemented in the clinical routine setting so far. PET in combination with MRI measures are increasingly being used to investigate new targets for measurement of acute and chronic inflammation and their consequences. A novel and intriguing approach uses PET-tracers focusing on the synaptic vesicle protein 2a (SV2A) [41].

 Gavilanes and colleagues investigated the performance of [^18^F]UCB-H to detect synaptic pathology in MS patients [42]. Using a mouse model, it was conclusively demonstrated that [^18^F]UCB-H is able to detect the synaptic loss in cortical grey matter lesions. In a second step, the authors applied this tracer in vivo to 32 MS patients with variable disease stages showing that SV2A-PET was able to detect substantial synaptic loss particularly in cortical grey matter lesions detected by high resolution brain MRI. In addition, the tissue volume of synaptic loss assessed by SV2A-PET was 20-fold larger compared to the cortical grey matter lesion detected by MRI. The observation by Gavilanes and colleagues that synaptic loss measures by [^18^F]UCB-H PET is more prominent in progressive MS patients further stresses the fact disease progression is associated with significant cortical grey matter pathology. Pending larger confirmatory studies, [^18^F]UCB-H PET aiming to measure synaptic pathology related to SV2A may be a promising target to assess and monitor cortical pathology and disease progression. 

 The synaptic vesicle protein 2A as a target to detect neuronal damage in MS has been also investigated by Luoma et colleagues using PET tracer [^11^C]UCB-J in addition to conventional MRI measures, such lesion based on T1 and T2-weighted FLAIR images [43]. This in vivo study enrolling 10 MS patients showed that MS patients compared to healthy control subjects had a significantly lower SVA2A availability in the cortical grey matter in all investigated topographies particularly in the temporal and insular areas as well as in deep grey matter structures. The fact that the SVA2A availability did not correlate with clinical outcome measures is most like due to the small sample size. Interestingly, it did also not correlate with the grey matter volumes. Unfortunately, the relation between cortical lesions and the tracer uptake has not been investigated in this study. Given the fact that the [^11^C]UCB-J has high specificity and sensitivity in detecting SV2A as well as good test-retest reproducibility, future studies using this tracer should be performed to correlate synaptic density with cortical grey matter pathology on high resolution MRI [44,45]. 

### Biomarkers in presymptomatic MS

There are several clinical, imaging and fluid biomarkers that are used for prognostic classification of MS patients [46]. Fissolo and colleagues investigated potential prognostic factors for development of MS symptoms in a multicentric cohort of 273 RIS patients with a focus on serum and CSF soluble biomarkers [47]. In their analysis, the presence of CSF immunoglobulin G (IgG) (HR 5,1) and IgM (HR 2,6) as well as a kappa free light chain index of above 6.1 (HR 2,8) were each associated with an increased risk of developing MS in these individuals. Furthermore, high CSF levels of neurofilament (HR 1,3) as well as high sNfl z scores (HR 1,4) were associated with an increased risk regarding the conversion to MS. The combination of two of these predictive variables, i.e. IgG oligoclonal bands and sNfl z scores conferred a 5-year risk of MS of 58,3 %, and this risk further increased to 81,6 % in the subgroup of younger patients up to 37 years. These findings are highly relevant as they point to the relevance of CSF analysis beyond increasing diagnostic accuracy in patients with of suspected MS. In addition, it illustrates how established CSF biomarkers can be utilized in people with a RIS constellation to select those who may particularly profit from early immunotherapy to delay MS symptom onset.

### Biomarkers to predict MS disease trajectories

Several new studies elucidated the relevance of Nfl and GFAP and their prognostic potential for distinct disease trajectories in MS. One of them investigated the relationship between demyelination and neuroaxonal injury using Nfl across rodent models and human cohorts [48]. The researchers demonstrated that Nfl levels peak during acute inflammatory demyelination, and – as expected – correlated with neuroaxonal damage. Notably, in a mouse model of inducible non-inflammatory demyelination, Nfl levels also rise during primary demyelination and decrease upon spontaneous remyelination. These findings were validated in human MS cohorts, were elevated levels of MOG as well as prolonged visual evoked potential latencies as surrogate parameters of myelin damage were robustly associated with increased blood Nfl. These data further establish Nfl as a sensitive marker of demyelination-associated neuroaxonal damage, for example during focal lesion development. Another study by Abdelhak and colleagues investigated the prognostic utility of the astrocyte marker GFAP and Nfl for predicting confirmed disability worsening (CDW) in progressive MS in a multinational effort encompassing 1058 participants [49]. In their population, median GFAP Z-scores (0.74) and median Nfl Z-scores (0.64) were higher than the theoretical healthy population Z-score of 0. They observed that higher baseline adjusted GFAP z-scores were significantly associated with a roughly 10 % increased risk of future CDW per z-score unit in the overall population, and this effect was primarily driven by the SPMS subcohort but not PPMS. In contrast, Nfl z-scores were predictive of disability progression in the PPMS subcohort, but not in SPMS. These data further support the notion that GFAP and Nfl are associated with distinct mechanisms of disease progression, and that these mechanisms seem to be somewhat distinct in SPMS and PPMS cohorts. In a similar fashion, another study by Maceski and colleagues investigated the complementary prognostic values of GFAP and Nfl for prediction of progression in a fingolimod-treated MS cohort [50]. They observed that elevated GFAP z scores were associated with a 1.64-fold increased risk of PIRA and further correlated with future cortical grey matter atrophy. In contrast, elevated sNfl specifically predicted future relapse activity. Another study by Brummer and colleagues addressed the prognostic utility of these two biomarkers for disease progression in a cohort of early MS patients who appeared clinically and radiologically stable [51]. Notably, in this setting, sGFAP levels showed no significant association with clinical outcomes, whereas baseline sNfl z-scores were significantly elevated in patients with increasing lesion volume who subsequently experienced EDSS progression. Multivariate logistic regression confirmed sNfl as an independent risk factor for disability in the subgroup of patients with increasing lesion volume. This suggests – taking the other studies into consideration - that in these early MS patients, disease progression may be primarily driven by insidious focal inflammatory activity and this is better captured by Nfl but not by GFAP. Finally, a study by Willard and colleagues utilized an unsupervised machine learning framework to integrate MRI-derived metrics with serum Nfl levels to characterize MS phenotypes [52]. Using a training cohort of 189 and an external validation cohort of 445 MS patients, they describe two biologically distinct trajectories – an "early-Nfl" subtype with rapid Nfl elevation associated with early lesion accrual and corpus callosum injury, and a "late-Nfl" subtype with early volumetric loss in deep grey matter areas accompanied by sNfl elevation at more advanced disease stages. They postulate that the latter subtype may indicate a more insidious neurodegenerative process preceding overt axonal injury.

In summary, these new studies further substantiate the predictive values of sNfl and GFAP for MS disease trajectories enforcing the concept that sNfl elevations are primarily associated with neuroaxonal damage in the context of inflammatory demyelination and therefore predict progression driven by inflammatory lesion development. GFAP on the other hand may predict disease progression primarily in constellations with an underlying pathobiology distinct from focal inflammatory activity, which has yet to be determined.

## Topic 9: Prediction of disease evolution using machine learning

In order to leverage new information out of existing data sets, machine learning algorithms are now employed in clinical MS research to evaluate and rank a predefined set of clinical imaging and demographic variables by quantifying their individual and combined contributions to a predictive model, thereby aiming at identifying which outcome parameters most strongly drive a specific phenotype. Two seminal papers published in 2025 used such ML approaches to facilitate prediction of disability trajectories in MS and ultimately guide personalized treatment decisions. In a nutshell, they provide evidence that machine learning can help to make use of well-known parameters gathered in clinical routine to learn more about a patients’ disease trajectories.

The study by Tur and colleagues investigated a clinically well-characterized monocentric prospective cohort of 1074 CIS patients and determined the prognostic value of distinct parameters to predict a disability milestone of an EDSS of 3.0 [53]. These parameters included demographic and clinical data, brain and spinal cord MRI, as well as CSF data. A survival random forest analysis identified four distinct subsets of patients with different disability trajectories over 20 years out of these conventional baseline parameters. The predictors in that model that carried the highest informative weight for reaching an EDSS of 3 were age at first attack, the number of T2 brain lesions at baseline, and baseline disability measured by the EDSS confirming the finding of earlier studies. Interestingly, use of these three parameters together already achieved a relatively high predictive accuracy of 0.72, even when more complex data were unavailable. The model performed even better when other parameters were included (up to an accuracy of 0.79), such as male sex, OCB and infratentorial involvement. This study has two important implications: First, from a therapeutic view, standardized assessment of this Barcelona risk score during first decision making for an initial immunotherapy may provide further information for guiding more personalized treatment decisions by incorporating a patient's individual disability trajectory. Second, it shows that well known and established parameters still are of increasingly prognostic value for patients, when their potential is leveraged by combination with new statistical approaches – in other words, new bioinformatic approaches help us to learn more from already existing data and knowledge.

The second study by Ganjgahi et al present a data-driven reclassification of multiple sclerosis disease trajectories based on data input from over 8000 patients collected in the NO.MS clinical trial data base and subsequently validated in an additional data set from 4000 patients from independent clinical and real-world cohorts [37]. On these data sets, they applied a probabilistic machine learning model, and move from traditional clinical descriptions (RRMS, SPMS; PPMS) towards a disease continuum defined by four dimensions: physical disability, brain damage, relapse, and subclinical radiological activity. These dimensions together give rise to eight discrete stages that were grouped into four clinical meta-stages of an MS disease continuum, i.e. early/mild/evolving MS, asymptomatic radiological activity, relapse, and advanced MS. The seminal finding of this study is that transition from the early stage to the advanced stage occurs almost exclusively through two inflammatory stages – either over relapses or subclinical radiological activity – rather than through direct progression. This finding implies that successful therapeutic control of any focal inflammatory disease activity represents a potent strategy to prevent transition of patients into advanced disease stages. For clinicians, the lesson from such a study is that rigorous control of treatment success and early treatment optimization in case of ongoing disease activity under treatment have to be even further prioritized to minimize patients' risk to transition into advanced disease stages. An open albeit highly interesting question is the relative performance of our available DMTs in prevention of patients transitioning towards the more advanced disease stages.

## Topic 10: New treatment approaches

In the landscape of emerging therapeutic strategies, current efforts are centered on optimizing treatment options for disease progression in multiple sclerosis (MS). A key therapeutic paradigm discussed at the 2025 ECTRIMS Congress investigated the impact of intensified B-cell depletion through the administration of high-dose of ocrelizumab in patients with primary progressive multiple sclerosis (PPMS) [54]. The rationale behind this approach was derived from a post-hoc analysis of the OPERA trials, which suggested a positive correlation between anti-CD20 antibody plasma levels and clinical efficacy regarding the mitigation of disability progression. Ultimately, however, the expectations for superior clinical outcomes through higher dosing of ocrelizumab investigated in the GAVOTTE clinical trial were not met [55].

Nevertheless, conceptually B-cells continue to be regarded as relevant drivers of disease progression. However, it appears that increased peripheral B-cell depletion is insufficient to achieve the necessary compartment-specific depletion of those B-cells sequestered behind the blood-brain barrier. Consequently, current clinical trials are focusing on enhancing the CNS penetrance of B cell depleting antibodies as well as exploring alternative mechanisms for B-cell eradication. These include bispecific antibodies equipped with specialized "brain shuttle" mechanisms, the application of CAR T-cell therapies directed against B-cell antigens, where CAR T cells achieve high tissue penetrance even within the CNS, as well as modulation of B cell activity by BTK (Bruton's tyrosine kinase) inhibitors. In this regard, in 2025 and beginning of 2026, novel clinical trial results have been presented on different Bruton's tyrosine kinase inhibitors (BTKis) in MS, BTKi holding promise of modulating adaptive and innate immunity by targeting both B-cell and myeloid lineages, including macrophages and resident microglia in case of sufficient BBB penetrance [56]. The high hopes into BTKis are driven by the concept that this class is uniquely positioned to address key aspects of compartmentalized inflammation driving disease progression by CNS located memory B cells and microglial cells. Following the failure of evobrutinib to demonstrate superiority over teriflunomide in earlier trials published in 2024, clinical trial data on the BTK inhibitor tolebrutinib were available in 2025. Results from the GEMINI trials in relapsing MS (RMS) showed that while tolebrutinib did not outperform teriflunomide in reducing annualized relapse rates, it exhibited a significant impact on disability progression in the pooled analysis of both parallel trials [57]. This was further validated in the HERCULES trial, where tolebrutinib achieved its primary endpoint by significantly slowing disability progression compared to placebo in patients with non-relapsing secondary progressive MS (nrSPMS) [58]. The clinical efficacy in the SPMS population in combination with the observed association between presence of baseline paramagnetic rim lesions (PRLs) as assumed *in vivo* correlates of chronic active inflammatory lesions and the extent of tolebrutinib-related reduction in disease progression—fueled high expectations for the PERSEUS trial in primary progressive MS (PPMS) [59]. However, data presented at ACTRIMS 2026 revealed that tolebrutinib failed to reach significance against placebo regarding disability progression in PPMS, despite a similar prevalence of PRLs at baseline in this cohort compared to HERCULES [60]. This discrepancy may at least be attributed to differences in inclusion criteria: the HERCULES trial required evidence of recent inflammatory disease activity illustrated by substantial documented clinical progression, whereas the PERSEUS cohort likely represented a population with lower overall inflammatory drive, potentially limiting the drug's observable impact. Another potential confounding aspect may be carry-over effects of previous B cell depletion in patients from the PPMS trial cohort.

Concurrently, at ACTRIMS 2026 congress, the FENtrepid trial data for fenebrutinib in PPMS were presented, where fenebrutinib demonstrated non-inferiority to ocrelizumab concerning disability progression in PPMS, reinforcing the biological viability of the BTK principle in this phenotype. Furthermore, very recently at the AAN 2026, the effect of fenebrutinib in RMS was presented based on data from the two FENhance trials, which stated significant reductions in relapse rates by fenebrutinib compared to teriflunomide (51 % reduction in FENhance 1 and 59 % reduction in FENhance 2), a result distinct from other investigated BTKis so far [61]. However, pooled analyses regarding disability progression showed only a favorable trend but did not reach statistical significance [61]. Ultimately, while this update confirms that BTKis remain a compelling strategy for addressing disease progression in MS, the heterogeneous outcomes across different molecules and patient populations suggest that the precise relationship between the cellular effects of BTK inhibition in MS, the relation between their peripheral and central effects, as well as disease subtype-specific response rates due to distinct underlying pathophysiologies still remains incompletely understood.

## Outlook

The successful prevention of lesion formation by immunomodulatory therapies has confirmed the pivotal role of the peripheral immune response in the development of focal lesions. However, it has also revealed the presence of additional pathophysiological processes beyond focal lesions that drive disease progression and contribute to the accumulation of disability. The studies selected here elucidate certain aspects of the underlying pathophysiology; nevertheless, the relative contributions of distinct cell types, entry routes, and molecular mechanisms to the individual disease course still remain to be solved.

A central challenge lies in the discrepancy between the complexity and heterogeneity of the disease and the limited tools currently available to comprehensively assess its multifaceted aspects in vivo at the level of the individual patient. Another major obstacle is the restricted transferability between post-mortem tissue-based omics studies and currently available in vivo biomarkers, largely due to the absence of systematic longitudinal investigations evaluating these diverse parameters within the same individuals.

Since the establishment of such a longitudinal cohort, including post-mortem tissue samples, is unlikely to be feasible in the foreseeable future, it becomes all the more imperative to optimize alternative research strategies. Accordingly, future large-scale clinical studies should strive to integrate comprehensive imaging modalities with diverse biomarker assessments and systematically archive biological specimens, thereby enabling subsequent investigations to disentangle the relative contributions of these pathomechanisms at the individual patient level through emerging biomarkers and advanced computational methodologies.

## Conflict of interest statement

L.K. received compensation for serving on scientific advisory boards and speaker honoraria from Alexion, Amgen, Argenx, Bayer, Biogen, Bristol-Myers Squibb, Grifols, Hexal, Horizon, Janssen, Merck Serono, Novartis, Roche, Sandoz, Sanofi, Santhera, Teva and Viatris.

T.K. received compensation for serving on scientific advisory boards from Novartis, Sanofi and Merck and speaker honoraria from Novartis, Biogen, Sanofi and Roche.
